# The gene expression profiles of primary and metastatic melanoma yields a transition point of tumor progression and metastasis

**DOI:** 10.1186/1755-8794-1-13

**Published:** 2008-04-28

**Authors:** Adam I Riker, Steven A Enkemann, Oystein Fodstad, Suhu Liu, Suping Ren, Christopher Morris, Yaguang Xi, Paul Howell, Brandon Metge, Rajeev S Samant, Lalita A Shevde, Wenbin Li, Steven Eschrich, Adil Daud, Jingfang Ju, Jaime Matta

**Affiliations:** 1 Mitchell Cancer Institute-University of South Alabama, 315 North University Boulevard, MSB 2015, Mobile, Alabama 36688, USA; 2 H. Lee Moffitt Cancer Center and Research Institute, 12902 Magnolia Drive, Tampa, Florida 33612, USA; 3 Xuanwu Hospital, 45 Changchu St., Xuanwu District, Beijing, 10053, PRoC; 4 Department of Pharmacology, Physiology and Toxicology, Ponce School of Medicine. P.O. Box 7004, Ponce, 00732, Puerto Rico

## Abstract

**Background:**

The process of malignant transformation, progression and metastasis of melanoma is poorly understood. Gene expression profiling of human cancer has allowed for a unique insight into the genes that are involved in these processes. Thus, we have attempted to utilize this approach through the analysis of a series of primary, non-metastatic cutaneous tumors and metastatic melanoma samples.

**Methods:**

We have utilized gene microarray analysis and a variety of molecular techniques to compare 40 metastatic melanoma (MM) samples, composed of 22 bulky, macroscopic (replaced) lymph node metastases, 16 subcutaneous and 2 distant metastases (adrenal and brain), to 42 primary cutaneous cancers, comprised of 16 melanoma, 11 squamous cell, 15 basal cell skin cancers. A Human Genome U133 Plus 2.0 array from Affymetrix, Inc. was utilized for each sample. A variety of statistical software, including the Affymetrix MAS 5.0 analysis software, was utilized to compare primary cancers to metastatic melanomas. Separate analyses were performed to directly compare only primary melanoma to metastatic melanoma samples. The expression levels of putative oncogenes and tumor suppressor genes were analyzed by semi- and real-time quantitative RT-PCR (qPCR) and Western blot analysis was performed on select genes.

**Results:**

We find that primary basal cell carcinomas, squamous cell carcinomas and thin melanomas express dramatically higher levels of many genes, including *SPRR1A/B*, *KRT16/17*, *CD24*, *LOR*, *GATA3*, *MUC15*, and *TMPRSS4*, than metastatic melanoma. In contrast, the metastatic melanomas express higher levels of genes such as *MAGE*, *GPR19*, *BCL2A1*, *MMP14*, *SOX5*, *BUB1*, *RGS20*, and more. The transition from non-metastatic expression levels to metastatic expression levels occurs as melanoma tumors thicken. We further evaluated primary melanomas of varying Breslow's tumor thickness to determine that the transition in expression occurs at different thicknesses for different genes suggesting that the "transition zone" represents a critical time for the emergence of the metastatic phenotype. Several putative tumor oncogenes (*SPP-1*, *MITF*, *CITED-1*, *GDF-15*, *c-Met*, *HOX *loci) and suppressor genes (*PITX-1*, *CST-6*, *PDGFRL*, *DSC-3*, *POU2F3*, *CLCA2*, *ST7L*), were identified and validated by quantitative PCR as changing expression during this transition period. These are strong candidates for genes involved in the progression or suppression of the metastatic phenotype.

**Conclusion:**

The gene expression profiling of primary, non-metastatic cutaneous tumors and metastatic melanoma has resulted in the identification of several genes that may be centrally involved in the progression and metastatic potential of melanoma. This has very important implications as we continue to develop an improved understanding of the metastatic process, allowing us to identify specific genes for prognostic markers and possibly for targeted therapeutic approaches.

## Background

In the United States, the overall incidence of melanoma is increasing at a rate faster than any other cancer, with recent estimates for the lifetime risk of developing invasive melanoma at 1/49 [1]. Patients diagnosed with metastatic melanoma (AJCC stage IV) have an overall poor prognosis, and 6 out of every 7 deaths associated with all types of skin cancer are caused by metastatic melanoma [1-3]. Whereas patients with thin primary tumors are cured after appropriate surgical intervention, most patients with advanced disease do not respond to available therapies, currently limited to only 2 FDA approved agents to treat stage III and IV melanoma. For most cancers, earlier detection methods have the greatest impact on survival and further contributing to better cure rates, with targeted treatment regimens depending upon an understanding of the molecular profile of a tumor. Similarly, we must improve our understanding of the mechanisms that lead to progressive and metastatic disease, as this will most likely provide the greatest hope for patients in reducing the number of deaths due to metastatic melanoma (MM).

The development of melanoma begins with the malignant transformation of normal human epithelial melanocytes (NHEM) located within the basement membrane of the skin. However, the exact cellular mechanisms that occur and the genes and molecular pathways involved in this process remain obscure. Early work has shown a direct correlation between the thickness of the primary cutaneous melanoma (PCM) and its metastatic capacity, either via the lymphatic system or hematogenously [4,5]. Once melanoma has metastasized by either route, the overall survival for patients greatly diminishes [6,7].

Many investigators have examined the differences between primary melanoma and the late stage metastatic disease. In the last few years, the power of microarray technology has been utilized to survey transcriptional differences that might provide insight into the metastatic process [8-15]. The earliest reports used a handful of samples to first suggest that MM was characterized by a large number of gene expression differences from cultured melanocytes or benign nevi [8,16]. Since then, many reports have corroborated this notion in principle, although each study has focused on a different aspect of the disease process. Some studies have used cell lines to compare melanocytes to melanoma [8,12], while others have utilized tissue samples to look at melanoma samples relative to benign nevi or normal skin [10,11,15,16]. Several studies have focused on the clinical aspects of the disease, attempting to identify gene expression signatures that correlate with metastasis or survival [9,13,17-19]. Together, these studies suggest that many genes contribute to the signature that is detectable in patients with MM. We wished to further examine the exact timing of when these gene expression patterns arise.

A few studies have begun to suggest that the metastatic expression pattern emerges during the vertical growth phase of primary melanomas. Initially, Smith *et al*. (2005) [10] showed a distinct transition point of gene expression change compared to normal skin, benign nevi, melanoma in situ (MIS), PCM, and MM samples. However, they did not specifically describe the Breslow's tumor thickness of their primary melanoma samples. More recently, Soikkeli *et al*. [19] in studying the draining lymph nodes for melanoma, concluded that many of the "molecular traits" of micrometastases were already present in primary melanoma. The Melanoma Group of the European Organization for Research and Treatment of Cancer (EORTC) has recently reported that melanoma patients with an average tumor thickness of 2.0 mm had a favorable prognosis whereas patients with thicker tumors (5.3 mm) faired worse [13]. Although no comparisons were made to patients with metastatic disease, this study reported a 254-gene classifier that significantly correlated with the metastatic dissemination of cutaneous melanoma. Similarly, Jaeger *et al*. [14] showed that different primary tumors could be grouped based upon gene expression.

In the current study, we have attempted to identify gene expression patterns that segregate non-metastatic tumors from MM, further examining thin PCM tumors to document the emergence of the metastatic signature. Additionally, we attempt to identify key genes involved in this process, as opposed to those genes that simply document the metastatic profile of a melanoma cell.

## Methods

### Tissue specimens

Over a 3 year period we surgically procured tumor samples from patients with primary cutaneous melanoma (PCM), squamous cell carcinoma (SCC), basal cell carcinoma (BCC) and metastatic melanoma (MM). All samples were obtained under an Investigational Review Board (IRB) approved tissue procurement protocol (MCC#13448, IRB#101751; PSM# 990914-JM, 020318-JM). Upon surgical removal of the primary melanoma, a single surgical oncologist (A.I.R.) utilized a scalpel to macrodissect and procure a portion of each tumor, careful to avoid central areas of necrosis and surrounding stroma. With bulky melanoma tumors, macrodissection usually results in the isolation of 90–95% pure tumor cells, with little interdigitating stroma or other contaminating tissues.

All samples for this study were confirmed to contain > 95% melanoma cells by a dermatopathologist. All samples were cryopreserved in liquid nitrogen and securely de-identified through a centralized database. We analyzed 40 MM samples, composed of 22 bulky, macroscopic (replaced) lymph node metastases, 16 subcutaneous and 2 solid organ metastases (adrenal and brain), and compared them with 42 primary cutaneous cancers [16 PCM, 11 SCC, 15 BCC]. PCM consisted of 2 melanoma in situ (MIS), 2 thin melanomas (< 1 mm), 3 intermediate-thickness melanomas (1–4 mm), and 9 thick melanomas (> 4 mm). Additionally, we included 4 samples of normal human skin and 1 sample of cultured, intermediate-pigmented, human epithelial melanocytes (NHEM).

### RNA isolation, purification and hybridization

A portion of each cryopreserved tissue sample was dissolved in TRIzol^® ^(Invitrogen, Carlsbad, CA), purified according to manufacturer's recommendations, and further purified on RNeasy columns (Qiagen Inc., Valencia, CA). RNA integrity was verified by both gel electrophoresis and the Agilent 2100 Bioanalyzer (Agilent Technologies, Palo Alto, CA). A total of 5 ug of RNA was processed using established Affymetrix protocols for the generation of biotin-labeled cRNA and the hybridization, staining, and scanning of arrays as outlined in the Affymetrix technical manuals [43,44]. The processed RNA was hybridized to Human Genome U133 Plus 2.0 arrays from Affymetrix, Inc. (Santa Clara, CA), and scanned on an Affymetrix GeneChip^® ^scanner 3000 at 2.5 μm resolution. A more complete description of this process is available in Dobbin *et al*., 2005 [45]. The tissue samples were processed in three independent groups.

### Cell lines and tissue culture

Freshly excised melanoma samples were placed into culture media (RPMI 1640 and 5% FCS) and tissue procurement and expansion of daughter cell lines was established utilizing previously published techniques [46,47]. All cell lines were passaged and expanded in vitro, utilizing RPMI 1640 and 5% FCS. All cell lines were split and passaged < 10 times and characterized by flow cytometry and cytospin preparations for cellular confirmation of melanoma cell purity (data not shown). The cell lines, MCC77 and MCC80a were derived from primary melanoma samples with TC80b derived from a metastatic lymph node (from the same patient). The cell lines, MCC12A and MCC12F, were derived from 2 different subcutaneous melanoma nodules from the same patient. There were 3 cell lines examined from metastatic samples, MCC66C, MCC72 and MCC89. The NHEM were cultured according to the manufacturer directions (Cambrex BioScience, Walkersville, MD). Several cell lines were obtained from the National Cancer Institute, Surgery Branch (624-Mel, 624.38-Mel and A375). These cell lines are derived from patients with stage IV melanoma and are considered highly aggressive cell lines by all standard measures of analysis.

### Real-time and semi-quantitative RT-PCR

First-strand cDNA synthesis was performed using Superscript III RT (Invitrogen). Subsequently, the cDNA was utilized for semi-quantitative PCR utilizing intron-spanning primers and optimized reaction conditions. We normalized each sample with β-Actin as an internal control, comparing each sample with AlphaEase^®^FC image analysis software (Alpha Innotech, San Leandro, CA), followed by densitometric analysis of the integrated values for each sample. The expression levels of putative oncogenes and tumor suppressor genes were analyzed by real-time quantitative RT-PCR (qPCR) using Assays-on-Demand Gene Expression Assays (Applied Biosystems, Foster City, CA): *SPP1 *(*osteopontin*, assay ID Hs00167093_m1), *GDF15 *(*growth differentiation factor 15*, assay ID Hs00171132_m1), *PITX1 *(*paired-like homeodomain transcription factor1*, assay ID Hs00267528_m1), *DSC3 *(*desmocollin 3*, assay ID Hs00170032_m1), *CST6 *(*cystatin E/M*, assay ID Hs00154599), *POU2F3 *(*POU domain, class 2, transcription factor 3*, assay ID Hs00205009) and *GAPDH *(assay IDHs99999905_m1) as the internal standard. Utilizing normal skin as the calibrator, the relative quantitation values of a target template for each sample were expressed as 2^-ΔΔCt^. Briefly, qPCR analysis was performed utilizing 40 ng of total cDNA in a 25 μl reaction volume (Applied Biosystems). We performed qPCR utilizing established techniques, with all samples performed in triplicate and run on an ABI/PRISM 7500 Sequence Detector System (Applied Biosystems).

### Gene microarray analysis and bioinformatics

MAS 5.0 analysis software was used to generate signal values for all probe sets based upon a mean intensity of 500, subsequently exported and iteratively normalized as a whole group to create the final normalization based upon the most stable gene expression measurements across all samples [48,49]. Genes highly expressed in metastatic melanomas but not in PCM, BCC, and SCC, or the converse, were specifically identified using visual inspection, t-tests and Pearson's correlation [50] [see Additional file 1]. An initial set of 2,014 Affymetrix probe sets were identified to discriminate between metastatic tumor samples and non-metastatic tumor samples and these were used for clustering. The normalized probe set values were log_2 _transformed and "mean-centered" across all clustered samples. Hierarchical clustering was then performed using absolute correlation and complete linkage in Eisen's cluster [51]. The complete microarray data is available from the Gene Expression Omnibus website [52].

Significance Analysis of Microarrays (SAM) was performed in order to identify a more extensive list of differentially expressed genes expressed between PCM and MM [53]. The first SAM analysis utilized all of the arrayed samples, with the MM samples opposed to all non-metastatic samples, inclusive of BCC, SCC and normal skin. A second SAM comparison utilized 6 thin PCM samples opposed to 6 MM samples. The median false discovery rate threshold was set at 5% for this comparison, with a final gene list generated by intersecting the two resulting gene lists. Following all microarray analyses, the identified probe sets were annotated based on the sequence of the probes used on the arrays [54].

### Western blot analysis

Whole cell extracts from PCM and MM cell lines were prepared by directly lysing cells in SDS sample buffer. Expression of SPP-1 protein was assessed in cell lysate and serum-free conditioned medium. Briefly, 4 × 10^6 ^cells were plated in 5% FBS containing medium; 24 hours later, the growth medium was replaced with serum-free medium. The conditioned media and cell lysates were harvested 24 hours later and resolved using a 12.5% SDS- PAGE. Proteins were transferred to a PVDF membrane and probed with the anti- human SPP-1 mouse monoclonal antibody (Sigma, St. Louis, MO) (1:1000) followed by a secondary antibody conjugated to horseradish peroxidase (Amersham Biosciences, Piscataway, NJ) and detected using chemiluminescence (Santacruz Biotechnology, Santa Cruz, CA). The osteopontin band (SPP-1) was visualized at ~55–65 kDa. Daughter melanoma cell lines derived from the freshly procured melanoma samples (with the exception of A375) were lysed by M-PER^® ^Mammalian Protein Extraction Reagent (Pierce, Rockford, IL) and processed according to manufacturer instructions. A total of 15 μg of protein from each experimental condition were electrophoresed on 10% SDS-PAGE and transferred to nitrocellulose membranes (Bio-Rad, Hercules, CA). Immunostaining was performed with the following primary antibodies: DSC3 (Santa Cruz) 1:200; CLCA2 (Novus Biologicals, Littleton, CO) 1:500; PDGFRL (Novus Biologicals) 1:500; α-tubulin (Cell signaling, Danvers, MA), 1:1000. Immunocomplexes were visualized using an enhanced chemiluminescence (ECL) Western Blotting Substrate (Pierce). The intensity of the bands were scanned with a Fujifilm intelligent dark box II and analyzed with Fujifilm Las-1000 Lite V1.3 software.

## Results

### Gene expression differences between primary cutaneous cancer and metastatic melanoma

An initial training set of 23 tumors revealed 2,014 Affymetrix probe sets with a greater than 2-fold difference in the average gene expression level between the MM and primary cutaneous cancers. This preliminary list, consisting of 1,141 well characterized and 471 poorly characterized human genes, was quite large, indicating a substantial difference between metastatic tumors and non-metastatic tumors. The expression differences allowed for a relatively robust gene classification of tissue samples into groups of metastatic samples and non-metastatic primary tumors. All tumor samples were clustered based on these gene expression values and individually identified as metastatic or non-metastatic based upon the characteristics of tumor samples in the same cluster.

The initial set of samples comprised a training set for which 22 of 23 samples were correctly partitioned into a cluster containing primary melanoma or the cluster containing MM samples. A single primary melanoma with a Breslow's tumor thickness of 90 mm was misclassified as a MM sample. We then analyzed and classified 2 independent test sets comprised of primary skin cancer (BCC, SCC and PCM) and MM samples, utilizing the same genes. Co-clustering led to the correct identification and classification of 56 of 60 melanoma samples. In general, the misidentified samples were thick primary melanomas classified as MM. Of note, several normal human skin samples were analyzed and found to classify as non-metastatic by their gene expression profiles.

We utilized all of the arrayed samples, exclusive of samples that were misclassified, in order to generate a more comprehensive list of genes that appeared to be differentially expressed between MM and PCM using SAM. This analysis identified 4,343 significantly different probe sets again indicating that malignant melanoma tumors are substantially different from the non-malignant tumors. All defined probe sets had an average difference greater than 2-fold between MM and PCM [see Additional file 2]. This list consisted of 1,667 Affymetrix probe sets that detect 279 poorly defined transcripts, 114 minimally defined genes, and 907 well characterized human genes. From this list, 303 genes were highly expressed in MM compared to 997 genes that were more highly expressed in the non-metastatic primary skin cancers and normal skin.

A subset of the full gene list is shown in Table 1, illustrating the trends in gene expression when MM samples are compared to *all *primary cutaneous cancers (BCC/SCC/PCM), or to only *very early *PCM (MIS/Thin), considered ≤ 1.5 mm in Breslow's thickness for this analysis. We found higher expression levels in MM for several genes implicated or known as melanoma-associated tumor antigens (*MAGE*, *CSAG2*) and some genes previously implicated in melanoma progression (*GDF15*, *MMP14*, *SPP-1*), cell cycle progression (*CDK2*, *TYMS*, *BUB1*) and the prevention of apoptosis (*BIRC5*, *BCL2A1*). Among the 997 genes with reduced expression in MM samples, several are implicated in keratinocyte differentiation and epidermal development, such as *loricrin *(*LOR*), *involucrin *(*IVL*), *keratin-5 *(*KRT5*) and *plakophilin *(*PKP1*), suggesting a loss of epidermal characteristics. The expression changes suggest important comparative differences between non-metastatic and metastatic tumors, with genes putatively defining cellular function as it relates to the metastatic process.

**Table 1:** Differential gene expression between metastatic melanoma and non-metastatic cutaneous tumors

**Gene symbol**	**Increase in metastatic melanoma relative to**		**Gene symbol**	**Decrease in metastatic melanoma relative to**	
	**MIS/Thin**	**BCC/SCC/MIS/Thin**		**MIS/Thin**	**BCC/SCC/MIS/Thin**
**MAGEA3/A6/A12**	125/119/57	27/25/29	**CALML5**	193	228
**MAGEA1/A2/A5**	24/57/10	13/31/6	**DSC1/3**	186/64	198/71
**CSAG2**	76	36	**PKP1**	166	240
**TRIM51**	51	35	**CLCA2**	162	177
**GDF15**	30	47	**DSG1/3**	160/73	178/119
**GYPC**	18	14	**LY6D**	143	147
**SPP1**	15	7	**SERPINB3/B5/B7**	111/199/106	184/227/144
**KIFC1**	15	3	**C19orf33**	122	135
**RGS20**	14	14	**FLG**	112	112
**C1orf90**	13	15	**KRT5/16/17**	49/111/105	62/196/274
**BCL2A1**	12	12	**KLK7/8/10/11**	99/23/27/65	112/32/81/83
**SOX5**	15	8	**LOR**	95	98
**SLC16A4**	12	29	**LGALS7**	84	89
**AKT3**	11	9	**CST6**	82	56
**PEG10**	11	10	**TRIM29**	79	119
**BUB1**	14	3	**SFN**	77	125
**RASGRF1**	8	12	**ASAH3**	69	56
**MMP14**	8	6	**GATA3**	63	54
**SPRED1**	6	4	**CBLC**	60	64
**GPR19**	6	5	**RAB25**	59	78
**CDK2**	6	7	**S100A14/A7/A7L1**	27/57/21	44/60/82
**HOXA10**	3	4	**ICEBERG**	52	48
**HOXB6/7/9**	4/5/3	6/7/3	**IVL**	50	76
**HEY1**	7	16	**ELOVL4**	38	34
**DUSP4**	8	10	**CXCL14**	36	37
**DUSP6**	8	6	**FOXN1**	33	34
**CDC45L**	7	8	**AQP3**	29	31
**CDC6**	9	4	**TP73L**	29	48
**RRM2**	6	4	**MUC15**	25	21
**TYMS**	4	3	**RORA**	24	22
**BIRC5**	4	2	**CD24**	19	38

The initial statistical analysis addressed the question of whether there were genes differentially expressed (increased or decreased) between primary cancers (BCC/SCC/PCM) and metastatic cancers (metastatic melanoma). Column 1 (MIS/thin) are comprised of only such lesions, while column 2 (BCC/SCC/MIS/Thin) is comprised of all 4 histologic subtypes. All of the annotated genes listed above had a > 2-fold up- or down-regulation in gene expression with the full names of each gene, accession number and gene identification provided in supplementary table 1. Abbreviations: MIS, melanoma-in-situ, BCC, basal cell carcinoma, SCC, squamous cell carcinoma, Thin, thin melanomas < 1.5 mm in Breslow's thickness.

A further analysis of the functional classes of genes changed using the gene ontology revealed that 15 genes associated with keratinocyte differentiation and 32 genes involved in epidermis development were down-regulated in the metastatic samples [see Additional file 3]. These losses were complemented by the increased expression of genes involved in several cellular processes, such as DNA repair, protein transport, melanocyte differentiation, muscle development, nervous system development and carbohydrate metabolism. Table 1 further illustrates that the magnitude of change in those genes under-expressed was much greater on average than the level of change in over-expressed genes. Overall, the losses in gene expression are both greater in number and magnitude compared to the gains in gene expression in MM samples.

### Identification of a transition point in gene expression between non-metastatic and metastatic tumor samples

**Figure 1:**
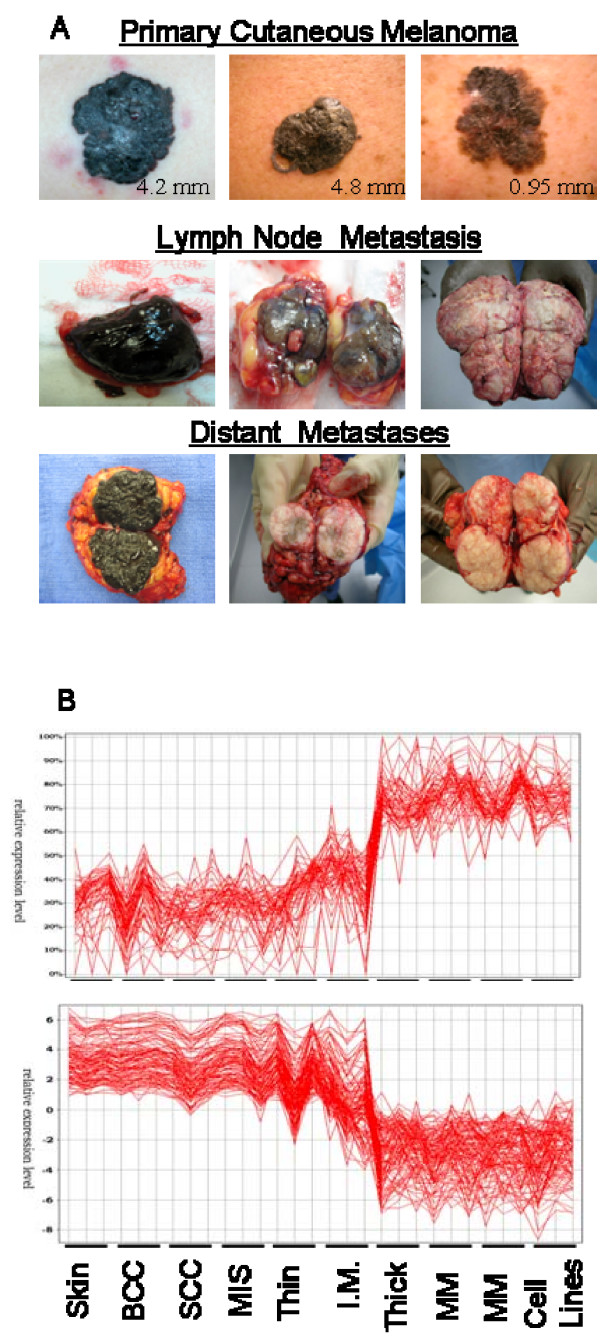
**Clinical primary and metastatic melanoma samples procured and cryopreserved at the time of surgery**. A: Intraoperative illustrations of the spectrum of PCM and MM samples procured. The PCM represents varying tumor thicknesses, measured utilizing Breslow's depth of invasion. All procured lymph node metastases were macroscopically involved, often completely replacing the entire lymph node parenchyma. Distant metastatic (subcutaneous and solid organ) melanoma often exhibited varying degrees of pigmentation, however, surrounding stroma was avoided in procurement of melanoma samples. B: A distinct change in the gene expression patterns is apparent within the comparative groups of thin/I.M. to thick PCM samples. Gene over-expression (upper graph) is evident at the I.M. thickness sample set, with an average Breslow's tumor thickness of 2.1 mm and 19 mm for thick melanomas. Contrary, there is a decrease in gene expression (lower graph) of the same set of genes, with a comparative difference in gene down-regulation evident at the same interphase of I.M. to thick PCM. Proceeding from left to right: normal skin, BCC, SCC, MIS, I.M., thick primary, metastatic melanoma (subcutaneous, lymph node and distant) and melanoma cell lines derived from patients with stage IV melanoma.

Figure 1B displays the comparative gene expression levels of 177 genes from our list across the spectrum of tissue samples examined, revealing a consistent level of expression through all of the presumed "non-metastatic" samples (normal skin, BCC, SCC, MIS, Thin). A marked change in the gene expression levels is seen, beginning with the I.M. thickness PCM (average Breslow's tumor thickness of 2.1 mm), progressively increasing or decreasing to the expression level representative of MM lesions and daughter MM cell lines. All of the thick PCM [average Breslow's thickness 19 mm] exhibited gene expression patterns similar to those of MM samples.

### Comparative analysis of gene expression patterns in primary melanomas of different Breslow's thickness

The apparent transition zone for gene expression could represent a critical time period where many tumorigenic events occur simultaneously or may simply reflect the outgrowth of an aggressive and/or metastatic cell phenotype. To address this issue, we evaluated the gene expression levels in PCM of increasing Breslow's thickness and that of MM samples. Table 2 (left columns) reveals the relative change in gene expression for a subset of genes throughout the spectrum of increasingly thick PCM and MM samples.

**Table 2:** Comparative analysis of gene expression changes in primary and metastatic melanoma.

**Gene Symbol**	**Relative increase in gene expression**					**Gene Symbol**	**Relative decrease in gene expression**				
	MIS to Thin	Thin To IM	IM to Thick	Thick to Met	MIS to Met		MIS to Thin	Thin To IM	IM to Thick	Thick to Met	MIS to Met

**MAGEA2**	<2	8.6	9.4	3.1	30.7	**SPRR1A**	<2	<2	<2	129.4	239.3
**MAGEA3**	<2	8.1	10.7	3	83.8	**SPRR1B**	<2	<2	<2	52.8	100.8
**MAGE A6**	<2	7.1	9.8	2.7	103	**KRT16**	<2	<2	<2	68.3	195.5
**MAGEA1,2**	<2	2.5	8.9	2.6	81.5	**KRT17**	<2	2.6	<2	27.4	57.6
**MAGE A1**	<2	<2	11.1	<2	25.4	**KRT6B**	<2	<2	<2	39.7	100.9
**MAGE A5**	<2	2.2	3.9	<2	11.7	**AQP3**	<2	<2	3.2	6.1	43.2
**MMP19**	5.4	<2	<2	<2	7.4	**CD24**	<2	<2	2	4.4	18.7
**PDGFRL**	22.3	3.6	<2	<2	6.3	**FLG**	<2	<2	3	24.1	140
**C16orf34**	5.4	<2	<2	<2	18.5	**IVL**	<2	2.2	<2	13.9	84.1
**CTH**	3.9	<2	<2	<2	8.8	**KLK7**	<2	<2	6.5	8.9	128
**GPR19**	4.2	<2	3	<2	23.9	**LGALS7**	<2	<2	3.4	17.3	109.3
**SPP1**	<2	13.1	<2	<2	44.9	**LOR**	<2	<2	2.4	22.6	120.4
**HOXA10**	<2	3.3	<2	<2	3.8	**RAB25**	<2	<2	3.3	10.3	88.4
**MMP14**	<2	3.6	<2	2.1	9	**SFN**	<2	<2	<2	10.6	24.2
**AKT3**	<2	<2	7.5	<2	14.3	**C19orf33**	3.1	<2	4.6	13.3	220.3
**BCL2A1**	<2	2.2	4.8	<2	18.7	**ASAH3**	<2	<2	25.9	<2	60
**BIRC5**	<2	<2	3.7	<2	3.4	**KRT15**	<2	<2	27.1	2.2	104.9
**BUB1**	<2	<2	9.4	<2	10.8	**ELOVL4**	<2	2.3	14.7	<2	41.9
**CDC45L**	<2	<2	13	<2	9.1	**GATA3**	2.5	<2	14.3	<2	23.5
**CDK2**	<2	<2	4.8	<2	8.4	**MUC15**	<2	2.2	11.9	<2	25
**CSAG2**	<2	<2	19.6	2.6	54.5	**SCEL**	<2	<2	28.8	<2	71.8
**DUSP4**	2	<2	5.7	<2	12.5	**TP73L**	<2	<2	5.5	3.4	41.2
**DUSP6**	<2	<2	3.7	<2	10.5	**RORA**	<2	<2	6.2	2.2	26.6
**GYPC**	<2	<2	12.2	<2	14.2	**POU2F3**	<2	<2	13.9	2.2	73.9
**HEY1**	<2	<2	5.7	<2	9.3	**ICEBERG**	<2	2.7	6.7	4.5	32.3
**KIFC1**	<2	<2	10.4	<2	16	**CASZ1**	<2	4.8	2.8	<2	12.3
**PEG10**	<2	2.4	4.1	<2	11.3	**HR**	<2	3.7	<2	<2	7.7
**RASGRF1**	<2	<2	5.1	<2	9.9	**TMPRSS4**	8.7	<2	<2	3.1	42
**RGS20**	3.4	<2	9.2	<2	34.3	**STAR**	4.9	<2	<2	<2	11.1
**SLC16A4**	<2	<2	4.4	<2	26.4	**ST7L**	4.3	<2	3.6	<2	8.4
**SOX5**	<2	<2	12.3	<2	19.8	**LTB4R**	4	<2	2.3	<2	9.9
**TRIM51**	<2	3.1	15.9	<2	63.6	**HAS3**	4.9	<2	<2	2.5	16.6
**TYMS**	<2	<2	3.6	<2	4.7	**FGFR3**	3.9	<2	3.2	<2	7.6

NOTE: All annotated genes listed above with a < 2 indicates that any difference between tumors for each comparative analysis was less than 2-fold. Underlined numbers indicate the greatest change in gene expression across varying PCM tumor thickness for each gene. Abbreviations: MIS, melanoma-in-situ, Thin, thin melanomas < 1.0 mm in Breslow's thickness, I.M., intermediate thickness between 1–4 mm, with thick melanomas > 4 mm. PCM, primary cutaneous melanoma.

Several genes, such as the *MAGE *genes, exhibited a steady and consistent increase in gene expression over the entire range of tumor thicknesses. However, we found a single major shift in expression for most genes when thinner primary tumors were directly compared to thicker ones. This was most apparent when comparing I.M. thickness to thick PCM, with the majority of genes showing the greatest comparative increase in gene expression during this period. Notable exceptions were genes such as *SPP1*, *HOXA10 *and *MMP14*, for which the greatest differential increase in expression was at the comparative interface between thin and I.M. thickness tumor samples. Other genes, such as *MMP19*, *CTH*, *PDGFRL*, *C16orf34 *and *GPR19*, showed the greatest comparative increase in expression when comparing MIS to thin PCM lesions.

We also found a similar phenomenon for genes with decreased expression in primary tumors relative to more advanced lesions (Table 2, right columns). Here, however, the largest proportion of the gene expression change occurred between thick PCM and MM samples. We found very little expression of *keratin *(*6B*, *16*, *17*) and *SPRR1 *(*A*, *B*) in MM compared to all primary melanomas, including thick lesions. Several genes, such as *TMPRSS4*, *STAR*, *ST7L*, *HAS3*, *FGFR3*, *CASZ1 *and *HR*, were found to have gene expression changes at the very earliest stages of tumor thickening. Together, the gene expression patterns do not shift in a coordinated fashion as would be expected as the result of the outgrowth of a clonal aggressive or metastatic cell type. Rather, the data suggests that a series of events occur as PCM tumors thicken that may ultimately influence the expression of different groups of genes. One limitation of this analysis is that only a few tumor samples were evaluated at the intermediate thicknesses, which allows the possibility that some of the observed differences might be tumor specific rather than stage specific.

### Comparative transcriptomic analysis of normal human epithelial melanocytes to primary and metastatic melanoma samples

We next compared the gene expression profiles of cultured NHEM to PCM and MM samples (Table 3), acknowledging the inherent limitations associated with the comparisons of cultured cells and freshly procured tumor samples [20]. We found large differences in gene expression when comparing NHEM to early, non-metastatic PCM (MIS/thin lesions only) and to MM samples suggesting that PCMs are already quite different from melanocytes. Genes such as *KRT14*, *GJA1*, *S100A7*, *S100A9*, and *EHF *were higher in most melanomas while genes such as *CITED-1*, *GDF15*, *QPRT*, *OCA2*, *c-MET *and *MME *were more highly expressed in NHEM. Many of the genes that increased (or decreased) in MM relative to thin PCM could be attributed to a shift toward expression levels similar to NHEM. For example, we found a decreased expression of keratinocyte proteins such as loricrin and involucrin. However, most of the melanoma associated antigens, including the *MAGE *genes, *PRAME*, *S100A8*, *TRAG3 *and *MMP19*, were more highly expressed in the MM samples than in NHEM.

**Table 3:** Comparative transcriptomic analysis of normal human epithelial melanocytes to thin primary cutaneous and metastatic melanoma samples

**Fold Higher Expression in Melanoma**				**Fold Lower Expression in Melanoma**			
**Gene Symbol**	**NHEM c/t MIS/Thin**	**Gene Symbol**	**NHEM c/t Metastatic Melanoma**	**Gene Symbol**	**NHEM c/t MIS/Thin**	**Gene Symbol**	**NHEM c/t Metastatic Melanoma**

**KRT14**	6787	**GJA1**	759	**MME**	106	**MAP4**	20
**GJA1**	5929	**SEPP1**	338	**CITED1**	77	**OCA2**	10
**EHF**	5487	**KRT14**	306	**GDF15**	64	**TRIM7**	7
**SCEL**	3931	**MAGEA2**	301	**PAEP**	47	**CITED1**	6
**CLCA2**	3689	**TRAG3**	242	**RPEL1**	45	**TRPM4**	6
**S100A7**	3609	**EHF**	193	**HES6**	43	**MME**	5
**KRTDAP**	3416	**S100A9**	160	**ESDN**	37	**TRAP150**	5
**DSC1**	2782	**S100A7**	129	**QPRT**	35	**FER1L3**	4
**GJB6**	2576	**SCEL**	126	**OCA2**	19	**QPRT**	4
**CXCL14**	2484	**SLC22A3**	125	**RENBP**	17	**KLF8**	4
**LOR**	2308	**EPHA3**	124	**NR4A3**	16	**RPEL1**	4
**KRT6A**	1989	**KRTDAP**	121	**Siat7c**	16	**PACE4**	4
**PKP1**	1835	**S100A8**	120	**C6orf168**	15	**HPCAL1**	4
**SERPINB3**	1778	**ZIC1**	119	**BCL2A1**	14	**ACTR1A**	4
**S100A9**	1768	**CXCL14**	118	**NTT73**	14	**MET**	3
**KRT15**	1545	**IL18**	108	**PSCD3**	14	**RAB32**	3
**GATA3**	1347	**PRAME**	102	**HPCAL1**	13	**TYR**	3
**PPL**	1339	**MAGEA6**	94.7	**MET**	12	**IRF6**	3
**IMUP**	1250	**PLCB4**	88	**ALS2CR3**	12	**GDF15**	3
**ICEBERG**	1123	**CLCA2**	87	**PTPLA**	12	**HINT3**	3
**KRT6B**	1114	**GJB6**	85	**TBC1D7**	11	**SLC30A1**	3
**CSTA**	693	**MAGEA3**	84	**OA1**	11	**TRPV2**	3
**CST6**	687	**MMP19**	59	**TYR**	11	**RSN**	3

NOTE: Abbreviations: NHEM: Normal Human Epithelial Melanocytes, c/t: Compared to, MIS, melanoma-in-situ, Thin, thin melanomas < 1.0 mm in Breslow's thickness

### Identification of putative oncogenes and tumor suppressor genes in melanoma

A perusal of the gene expression differences between PCM and MM samples identifies numerous putative oncogenes and tumor suppressor genes (TSG). Table 4 lists several of the known oncogenes and TSG previously implicated in tumor types. The gene with the largest increase in expression (13.2 fold) was *osteopontin *(*SPP1*). Although not previously identified as an oncogene, *osteopontin *expression has been shown to strongly correlate with melanoma invasion and tumor progression [21]. The lineage-specific oncogene, *MITF*, previously shown to act as a master regulator of melanocyte development and a critical survival oncogene amplified in melanoma, showed a 3.7 fold increase [22-24]. Of the other listed genes, *GDF15*, *c-Met *and the *HOX *loci have been shown to act as possible oncogenes in breast cancer, squamous cell lung cancer, prostate and pancreatic cancer. Several of the putative melanoma TSGs have also been previously shown to contribute to the development and progression of cancer in other tumor histologies.

**Table 4:** Differential expression of putative tumor oncogenes and suppressor genes in melanoma

**Oncogenes**				
**Gene**	**Fold Increase**	**Interval of Increase**	**Activating Mechanisms in other Tumor Histologies**	**Affected Tumor Types**

**SPP-1**	13.2	Thin to IM	C-Met activation via αvβ3 receptor; Inhibition of apoptosis	Breast, HCC, Prostate, CRC, Head & Neck
**MITF**	3.7	Progressive increase	Somatic alteration via gene amplification (Chr.#3p13-3p14)	None, Lineage Specific for Melanoma
**CITED-1 (cbp/p300 transactivator)**	12.4	IM to Thick	Activation of Stat-3, Ras/MAPK kinase signaling via Ets1, Ets2	Thyroid
**GDF15 (PLAB)**	22.7	IM to Thick	Lineage specific activation or repression of ERK1/2; Integrator of AKT pathway	Breast, CRC, Gastric, Prostate, Pancreatic
**c-Met**	14.5	Thick to Met	Ras-Associated Protein (Rap1)/ERK/MAPK, rac1, Grb2, PI3K, src activation	CRC, Breast, Ovarian, Pancreatic, Liver
**HOX Locus (A3, A10, B6, B7, B13)**	2.1 – 5.0	Progressive increase	Downstream activation of WT-1, NFKB, NR4A3, BCL2, p53	AML, Breast, SCLC

**Tumor Suppressor Genes**				

**Gene**	**Fold Decrease**	**Interval of Decrease**	**Suppressor Mechanisms in other Tumors Histologies**	**Affected Tumor Types**

**PITX-1**	13.9	Thin to IM	Ras Pathway (RASAL1)	Barrett's [Esophagus] Prostate, Bladder
**CST6 (CST E/M)**	66.7	IM to Thick	Hypermethylation	Breast, Glioma
**PDGFRL**	7.3	IM to Thick	Gene Deletion from Chr.# 8p21.3-p22	HCC, CRC, NSCLC
**DSC3**	42.8	Progressive decrease	Hypermethylation	Breast
**POU2F3**	49	Thin to IM	Hypermethylation	Cervical
**CLCA2**	162	MIS/Thin to MM	Hypermethylation	Breast

Note: Tables represent a partial list of identified tumor oncogenes and tumor suppressor genes (TSG's) in PCM and MM samples. The fold increase represents the greatest fold change noted throughout all comparisons of each PCM tumor thickness to MM. The activating/suppressive mechanism and affected tumor type are also identified. Abbreviations: HCC, hepatocellular carcinoma, CRC, colorectal carcinoma, NSCLC, non-small cell lung cancer, SCLC, squamous cell lung cancer, AML, acute myelogenous leukemia.

The shifts in gene expression occur at different stages of the thickening process for each of the oncogenes and TSGs listed in table 4. Some of the genes show a progressive and steady increase (or decrease) in gene expression as tumors of greater thickness are compared. For other genes, such as *SPP-1*, *GDF15 *(putative oncogenes), *PITX-1 *and *CST6 *(putative TSG), the major shifts in gene expression appear to occur at distinct but different times during the thickening of the primary melanoma tumors. This observation suggests that these changes may occur spontaneously but eventually may accumulate to contribute to the final metastatic phenotype.

### Validation of select candidate genes by semi- and quantitative RT-qPCR analysis

**Figure 2:**
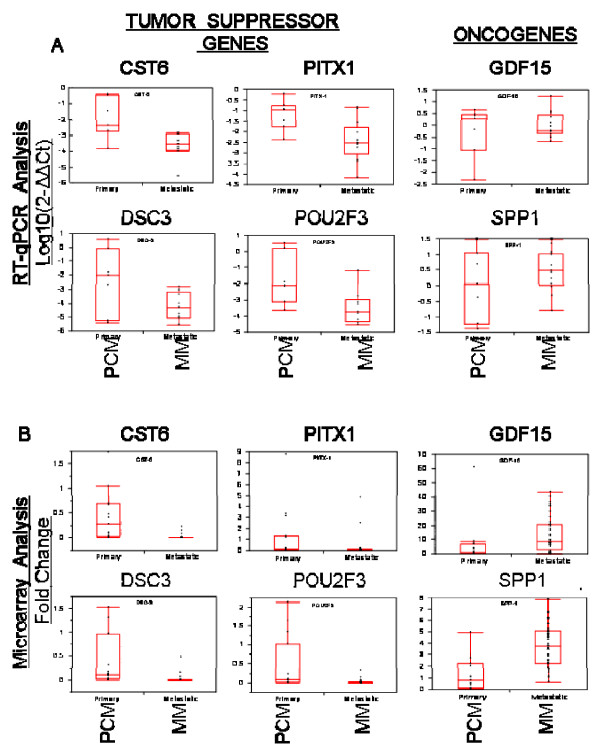
**Comparative gene expression analysis of putative oncogenes and tumor suppressor genes**. A: Comparative gene expression analysis utilizing qPCR data of putative tumor suppressor genes (CST6, DSC3, PITX1, POU2F3) and oncogenes (GDF15, SPP-1) in PCM (n = 7) and MM (n = 13) samples. Relative quantitation of target gene expression for each sample was determined using the equation 2^-ΔΔCt^, where GAPDH was used as the internal reference and normal skin as the calibrator. Values were Log base 10 transformed (y-axis) so that all values below zero represent a down-regulation in gene expression and values above zero represent an up-regulation in gene expression, compared to normal skin. B: Correlative microarray analysis of gene expression levels in primary and metastatic melanoma samples compared with normal skin. The statistical differences of gene expression between primary (PCM) and metastatic melanoma (MM) samples were analyzed by Wilcoxon's signed rank test; two-tailed significance level was set at α = 0.05. Compared to PCM samples (n = 7), the expression levels of 4 putative tumor suppressor genes (CST6, p < 0.0001; DSC3, p < 0.0001; PITX1, p = 0.0043, POU2F3, p < 0.0001) were significantly decreased in MM samples (n = 40), while the expression of putative oncogenes (GDF15, p = 0.0027; SPP1, p < 0.0001) were significantly increased in MM samples.

To further validate the expression of putative TSG and oncogenes in our melanoma panel, RT-qPCR was performed on 20 previously arrayed samples, comprised of 7 PCM and 13 MM samples. Figure 2A shows an overall decreased level of mRNA expression of TSG and increased mRNA expression of oncogenes compared to normal skin. This was consistent for all PCM samples compared to MM, although not statistically significant when comparing across Breslow's thicknesses. We then re-examined and compared the RT-qPCR data to the microarray data in order to validate the overall degree of agreement, finding a significant correlation for all TSG and oncogenes examined (Figure 2B).

**Figure 3:**
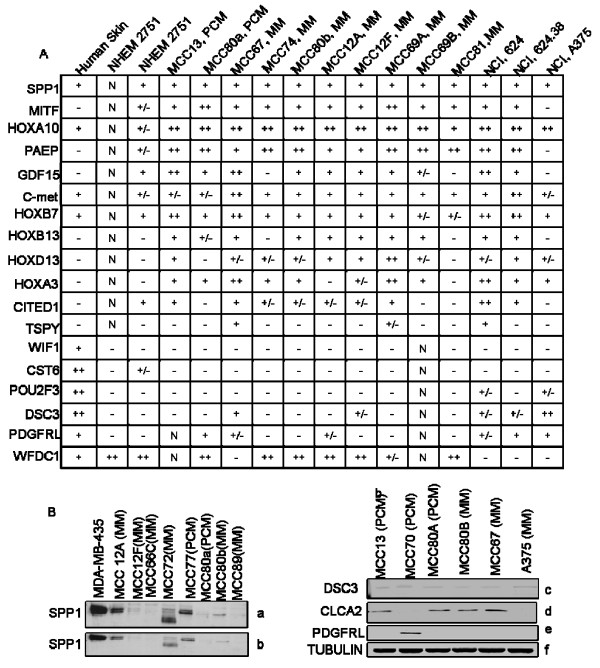
**Semi-quantitative RT-PCR analysis of oncogene and tumor suppressor genes**. A: A panel of 12 putative oncogenes and 6 TSG were analyzed, with the relative levels of mRNA expression as follows: negative band (-), faintly visible band (+/-), visible band (+), strongly visible band (++), N = Not Done. β-actin served as the internal comparative control. The grey values of PCR products of each gene are analyzed by the AlphaEase 3 software and standardized according to β-actin in every sample. B: a, b, Daughter melanoma cell lines secrete SPP1 (osteopontin). The melanoma cell lysates and conditioned cell-free media was resolved by SDS-PAGE and transferred to a PVDF membrane. The blot was probed with anti-SPP1 antibody. **c-f**, Antibodies for DSC-3 (c), CLCA2 (d), PDGFRL (e) and α-tubulin (f) as an internal control. Lanes 1–3 are PCM and lanes 4–6 are MM cell lines.

Utilizing semi-quantitative PCR analysis, we also examined several primary and MM daughter cell lines derived from the freshly procured melanoma samples, normal skin and NHEM for oncogene and TSG mRNA expression (Figure 3A). Consistent with the data obtained from microarray analysis, several of the melanoma derived cell lines exhibited high levels of expression for several of the reported oncogenes and a higher percentage of loss of TSG. Overall, we found a favorable correlation between the microarray results and both quantitative and semi-quantitative PCR analysis for daughter and non-daughter primary and MM cell lines.

### Validation of gene expression and protein translation of select candidate genes by Western Blot analysis and immunohistochemistry

To independently verify and validate the gene expression changes at the protein level, we studied several suspected oncogenes and TSG for protein expression by Western Blot analysis. Osteopontin (SPP1) protein expression was examined from melanoma cell lysates and conditioned cell free media derived from 2 primary and 6 MM daughter cell lines (Figure 3B, a, b). Interestingly, the protein expression level of 2 subcutaneous MM nodules (MCC12A, 12F) procured from the same patient greatly differed. Similarly, we examined 2 paired cell lines (MCC80a from a primary melanoma from a synchronous metastatic lymph node, MCC80b), showing a slight increase in SPP1 protein expression in the latter. Several other melanoma cell lines exhibited minimal SPP1 protein expression (MCC12F, 66C, 80a and 89), with comparable findings noted between melanoma cell lysates and conditioned cell media.

Analysis of suspected TSG in 3 PCM and 3 MM cell lines revealed a very low level of protein expression of DSC3 in 6/6 cell lines (3c) with 4/6 (2/3 primary and 2/3 MM) cell lines expressing the protein for CLCA2 (3d). Interestingly, we found PDGFRL protein expression in a single primary cell line, with no evidence of expression in any of the metastatic cell lines (3e). Lastly, we chose to utilize existing databases that have previously determined the cellular staining patterns utilizing immunohistochemistry in skin and melanoma samples, available for viewing at the Human Protein Atlas website [55]. This website also highlights those available genes with available immunohistochemical staining patterns of both primary and MM samples [see Additional file 2].

## Discussion

In this study, we have examined the gene expression profiles of a number of cutaneous tumors with the intention of highlighting the differences between metastatic tumors and non-metastatic tissues. We have profiled normal skin, melanocytes, BCC, SCC, and early stage melanomas to establish a gene expression pattern associated with the non- metastatic state. It is well known that BCC and SCC have little if any metastatic potential, and thus adding such specimens substantially contributed to the overall validity of our "non-metastatic" gene signature. Likewise, similar findings are noted for MIS lesions and very early primary melanomas (< 0.75 mm in Breslow's depth). Opposed to this is the metastatic state, which exhibits vastly different expression levels in thousands of genes and is observed in thick PCM and MM tumors. The principal finding of this study is that normal tissues and non-metastatic cutaneous tumors can be distinguished from metastatic melanoma tumors on the basis of the expression level of these genes. We have reported 1,300 transcripts that differ in expression between thin PCM and MM, many of which differ more than 100-fold. This robust signature suggests that gene expression may be useful for identifying metastatic potential in PCM tumors.

A number of previous studies have attempted to use gene expression signatures to identify the genes associated with the establishment or identification of metastatic tumors. Using cell lines it has been shown that normal melanocytes differ greatly from tumor derived cells [8,12]. Benign nevi have been shown to differ from thick or metastatic melanoma tumors [10,11]. Also, several recent studies have shown that MM or thicker melanoma tumors contain one or more distinctive signatures that may indicate the more aggressive phenotype with respect to clinical outcome [9,13,14,17-19]. Although each of these studies was unique in the samples they used and the gene expression signatures they report, we believe there is a very high degree of similarity between all of the "signatures".

The recent report by Jaeger *et al*. coincides very well with the current study [14]. Although they compared tumors to normal skin they utilized U133A arrays from Affymetrix, which contain a subset of the same probes found on the U133 Plus 2.0 arrays used in the present study. We compared the common probe sets of both arrays, finding a 65% overlap between the two gene lists. This is an exceedingly high level of concordance between different microarray studies utilizing tumor samples; probably reflecting the fact that the two studies used nearly identical microarray platforms.

There remains considerable debate in the literature as to why comparative microarray studies do not completely coincide with each other. We believe that the primary reason for such discrepancies is that strict p-values and other restrictive criteria are often utilized to generate gene lists. If we relax our criteria only slightly to generate a larger list than reported in supplementary table 1, we find a stunning 90% concordance with the study of Jaeger *et al*. The remaining differences between the two studies appear to be a few differences that exist between normal skin and metastatic melanoma rather than between primary melanoma and metastatic melanoma and a few genes that exist within a small subset of melanoma tumors and therefore most likely reflect subtle differences in the spectrum of tumors analyzed by the two studies (based on our data).

In all of the recent gene expression studies an attempt has been made to compare normal, benign, or non-metastatic cells or tissues to clinically aggressive tumors. In most of these studies, it is not clear where the shift in gene expression occurs. However, in the current study, we used a larger number and variety of non-metastatic tissues to illustrate the fact that the metastatic signature is robustly different from all the non-metastatic conditions that one might use for comparison. We have also included a documented gradation of tumor thicknesses to better resolve thin primary melanomas from thick primary melanomas which has helped to illustrate that the metastatic signature emerges as primary melanoma tumors thicken.

We have approximated what might occur in an individual tumor by grouping the melanoma samples into subgroups from thin through thick and proceeding on to metastatic tumors (Figure 1B). This illustration follows the analysis of Smith *et al*. [10] who compared normal skin, benign nevi, melanoma in situ (MIS) and MM samples to show a distinct transition point of gene expression associated with the vertical growth phase of melanoma tumors. However, that study did not specifically describe the Breslow's tumor thickness of their primary melanoma samples leaving open the question of what was metastatic and what was a non-metastatic tumor sample. We believe that our data resolves this issue, showing that MIS and thin melanomas do not have much, if any, of the gene expression measures associated with metastasis. The metastatic signature emerges when primary melanomas begin to thicken. This data suggests that thickening tumors is the best arena for further evaluating the cellular changes leading to metastasis.

Thin melanomas are the most difficult samples to acquire in a research setting where RNA integrity can be preserved and thus our analysis is limited by the few samples of thin melanoma we were able to collect during this study. Thus, it is probable that we have not identified the optimal progression in the transition from non-metastatic gene expression to metastatic gene expression. However, by looking more closely at the various thicknesses of tumors, we observed that the gene expression changes do not occur synchronously. Certain changes occur early and others appear later in the progression from thin to thick tumors (Table 2). Each change brings the expression pattern of primary melanoma tumors closer to the expression pattern observed in metastatic tumors. This suggests that the phenotype of tumors becomes more metastatic-like as the tumor gets thicker and argues against the outgrowth of a cell with the full metastatic signature.

Our data also indicates that many phenotypic changes may be occurring during this thickening period. Our gene ontology analysis suggests a fundamental shift in the functional properties of the cells comprising a tumor. There is a reduced expression or loss of genes involved in the processes of keratinocyte differentiation, epidermal development, cell adhesion and cell-to-cell signaling. This loss of cell-stromal interactions may reflect the gain of migratory potential for the metastatic cell type. This is opposed by functional gains associated with the increased expression of genes involved in melanocyte differentiation, nervous system development, protein transport, carbohydrate metabolism and DNA repair. The unidirectional shift in these classes of genes, the number of genes involved, and the extent to which each gene changed (fold-changes of 30 or more) all suggest a developmental change, rather than a regulation of cellular metabolism. Further supporting such a fundamental change is the observation by Alonso *et al*. that an epithelial-mesenchymal transition occurs as the metastatic signature emerges [18].

The thickening of PCM is also when the increased expression of putative proto- oncogenes and decreased expression of putative TSGs occurs (Table 3). Although most of these oncogenes and TSGs have not been demonstrated to function in melanoma progression, they represent key factors to consider when understanding the emergence of the metastatic phenotype. *Growth differentiation factor-15 *(*PLAB\MIC-1\GDF-15*) shows a striking correlation between expression level and metastatic phenotype in several tumor types [25,26]. Osteopontin (SPP-1) is a secreted phosphoglycoprotein that has been implicated in tumor progression and invasive behavior in many tumor types [27]. SPP-1 expression has been strongly correlated with invasive melanoma but found highly expressed in only 72% of invasive primary melanomas [21]. Our data shows that *SPP-1 *gene expression increases when thin primary melanomas thicken (Table 2) and that SPP-1 is expressed in daughter melanoma cell lines and secreted as a soluble protein (Figure 2). Other identified genes, such as *Cbp/p300-interacting transactivator *(*CITED-1*), *hepatocyte growth factor receptor *(*c-MET*), and various homeobox genes have yet to be investigated in melanoma although some have been previously identified in metastatic melanoma [11].

The TSGs listed in table 3 have been implicated in the evolution of the metastatic state via down-regulation and inactivation. *PITX-1 *has been identified as a TSG in several tumor types, including lung cancer and Barrett's esophagus leading to esophageal adenocarcinoma [28-30]. Loss of *PITX-1 *expression is seen in thin primary melanoma samples, with a 14-fold decrease in gene expression in thin primary compared to I.M. samples. Many of the other TSGs listed in table 3 have been down-regulated by epigenetic silencing in other tumor histologies [31-38]. Recently, Muthusamy *et al*. described the epigenetic silencing of novel tumor suppressors in melanoma samples, identifying 17 genes not previously known to be hypermethylated. [39]. We have found 2 genes that are in common with this list. We are actively examining the identified TSGs for evidence of epigenetic silencing via hypermethylation of promoter CpG islands.

We also found several known melanoma tumor antigens (*MAGE, TRAG3, PRAME*) highly expressed in thicker tumors. This is consistent with studies that have shown an increase in tumor antigen expression with advanced disease [8,40-42]. Some of these antigens were seen in nearly all tumor samples while others were only seen in a subset of the melanoma tumors. Nonetheless, these surface antigens provide a mechanism for identifying the metastatic cell arising in early melanoma tumors.

Based upon their distinct gene expression profiles, the four classes of tumors could be correctly classified greater than 90% of the time. The major difficulty in classification was for thick primary melanoma tumors that appeared to have the gene expression signature of MM tumors. The difficulty in classifying these tumors suggested that they might represent a transitional state between primary tumors and truly metastatic tumors. If we accept this assumption, then 81 of our 82 tumor samples were correctly classified based upon their gene expression signature. With the ability to correctly and accurately classify tumors into distinct classes, we can begin to investigate the mechanisms responsible for malignant tumor formation, invasion, progression and metastasis.

## Conclusion

Based upon our molecular analysis of primary cutaneous (BCC, SCC, PCM) and MM samples, we conclude that PCM and MM have distinct gene expression profiles that may be useful for tumor tissue classification. Additionally, squamous cell and basal cell carcinomas share certain gene expression patterns with primary melanomas that are distinct from MM. This is important to try and develop prognostic markers for patients based upon the distinct molecular profile of their melanoma, further able to distinguish between a metastatic versus non-metastatic gene profile. This will allow for the physician and patient to have a more informed discussion as to how to proceed with clinical treatment options based upon the gene expression profile.

## Authors' contributions

AIR is responsible for the initial conception, overall hypothesis and design of this manuscript, including the original draft and all subsequent revisions. AIR is also responsible for the procurement and cryopreservation of melanoma tissue specimens. SAE performed the molecular studies with gene microarray analysis as well as the bioinformatics data derived from all samples. SAE also assisted with the draft of this manuscript. JFJ and OF assisted with the draft of this manuscript and the numerous revisions and formatting for the final version. SL, SR, PH and BM all assisted with outstanding technical assistance, including the PCR analysis, western blotting experiments and performing all other confirmatory and functional data associated with this manuscript. All were also involved in the drafting and revisions for this manuscript. CM assisted with the statistical interpretations of the functional data. RSS and LAS both assisted with the Western blotting and cell analysis of SPP expression and were involved with the draft and revisions of this manuscript. WL was instrumental in the in vitro expansion of the melanoma cell lines and associated experiments with all daughter cell lines. JFJ and YX assisted with the semi- and quantitative PCR experiments and provided excellent guidance as to quality control for many of the experiments. SE provided the SAM analysis. AD provided some of the initial concepts and design for all subsequent experiments and was involved in the draft and revisions for this manuscript. JM assisted with the initial concept and design of the experiments and was also involved with the draft and revisions for this manuscript. All authors read and approved the final manuscript.

## Pre-publication history

The pre-publication history for this paper can be accessed here:



## Supplementary Material

Additional file 1 Detailed Gene Microarray Analysis and Bioinformatics. This document provides a more detailed and in-depth description of the statistical and biostatistical process involved in the supervised analysis of the generated data. We have also added a description of patient demographics. Click here for file

Additional file 2 Final Supervised Gene List. The data in this file describes the following: Supervised Gene list, symbol, GenBank reference ID #, Entrez Med ID #, UniGene ID #, Human Protein Atlas Tissue Staining Link for each gene. Click here for file

Additional file 3 Gene Ontology and Biological Processes. This file shows the ontological comparisons of various numbers of genes associated with each biological process between primary and metastatic melanoma. Click here for file
