# Supplementary material for: The gene expression profiles of primary and metastatic melanoma yields a transition point of tumor progression and metastasis
Source: BMC Med Genomics. 2008 Apr 28;1:13. doi: 10.1186/1755-8794-1-13 (PMC2408576; doi:10.1186/1755-8794-1-13)
Supplement: Additional file 1 — Detailed Gene Microarray Analysis and Bioinformatics. This document provides a more detailed and in-depth description of the statistical and biostatistical process involved in the supervised analysis of the generated data. We have also added a description of patient demographics. [file 1755-8794-1-13-S1.doc]

**Detailed Gene Microarray Analysis and Bioinformatics.**

The Affymetrix MAS 5.0 analysis software was used to generate Signal values for all probe sets in each array based on a trimmed mean intensity of 500 for each sample. The signal values from each array were then exported and all the arrays used in this study were iteratively normalized as a group (27, 28). This process insures that the final normalization is based on the most stable gene expression measurements across all samples. This process was performed for the initial group of tumor samples to generate the list of normalization probesets (supplemental data 4) which were subsequently used to scale all samples processed for this study to an average intensity of 4000 for the normalization probesets. Following scaling the calculated signal values were then used to calculate the average expression level for each gene in each tissue type using an initial group of 23 tumor samples.

We initially opposed the metastatic melanomas with the non-metastatic primary melanomas, basal cell carcinomas, or squamous cell carcinomas and used a t-test to identify potential genes differentially expressed between the two groups. From this list of genes we visually inspected the gene expression profiles across all the samples specifically looking for genes highly expressed in metastatic melanomas but not primary melanomas, basal cell carcinomas, or squamous cell carcinomas. Several genes were initially selected that exhibited this idealized gene expression profiles. Additional candidate genes were then identified by using Pearson’s correlation between the idealized gene expression patterns and all other probe sets on the arrays. Positively correlated (r >0.7) and negatively correlated (r <-0.7) genes were identified and this list of genes was trimmed to include only those with a 2-fold or greater difference in the average gene expression level between metastatic samples and non-metastatic tumors. This initial gene expression survey identified 2014 Affymetrix probe sets from the U133 Plus 2.0 arrays that showed differential expression between metastatic tumor samples and non-metastatic tumor samples.

The 2,014 probe sets identified as correlating with the metastatic phenotype were used to cluster the samples. Following normalization, as described above, the signal values were log_2_ transformed. Each probe set was then mean centered across all samples and the resulting values were input into Eisen’s cluster. Hierarchical clustering was performed using absolute correlation and a complete linkage. Clustering was performed with various subgroups of the data or with all samples together and resulted in similar sample groupings. Individual samples were classified based on the class of the other samples in the closest cluster. A similar method was employed to look for genes that would distinguish the BCCs from the SCC and the melanoma samples. Using the probesets found clustering was also performed to identify samples that were BCC or SCC rather than non-metastatic melanoma.

Serial analysis of microarrays (SAM) was performed to identify a more extensive list of genes differentially expressed between MM and PM. The SAM analysis made use of all the arrayed samples. Two comparisons were made to generate a comprehensive and yet confident list of genes that are differentially expressed between metastatic melanoma and non-metastatic melanomas. In the first comparison, the metastatic melanoma samples were opposed by all the non-metastatic samples including basal and squamous cell carcinoma and normal skin. The false discovery rate threshold used to limit the gene list was 0% for this comparison. Because of the number of samples, this provides good statistical confidence in the gene expression differences between non-metastatic and metastatic samples but does not focus on the differences specifically in melanoma.

A second comparison was therefore performed utilizing 6 thin primary melanoma samples in opposition to 6 selected metastatic melanomas from cutaneous tumors. Metastatic samples were selected to avoid choosing samples in which the classifier disagreed with the pathologist’s diagnosis and to avoid utilizing more than one sample from the same individual, otherwise the selection was random. This latter comparison will rule out differences due to tumor location and minimize differences of keratinocyte like tumors and melanomas. For this comparison the median false discovery rate threshold was set at 5%. This latter analysis is the preferred grouping of samples, but because of the small sample size it is also more likely to generate false discoveries due to noise and outlier samples. Therefore the more confident gene list is generated by combining the two analyses. The intersection of the two approaches yielded 1,352 probe sets with higher expression in the metastatic samples and 2,991 probe sets with higher expression in non-metastatic samples. This list was further reduced by removing probe sets that did not appear to have a difference greater than 2-fold on average between the two groups. The results of this analysis are presented in supplemental table 1. This final list consisted of 1667 Affymetrix probe sets that detect 279 poorly defined transcripts, 114 minimally defined genes, and 907 well characterized human genes. From this list 303 genes are more highly expressed in metastatic melanoma than non-metastatic cancers and 997 genes are more highly expressed in the non-metastatic cancers and normal skin.

Following all microarray analyses the identified probe sets were annotated based on the sequence of the probes used on the arrays (26). These annotations are also provided in supplemental table 1. All primary tumors identified by the attending physician and the pathologist were included in the non-metastatic melanoma class. For subgroup analysis we grouped PCM’s based upon Breslow’s thickness where: thin: <1 mm, intermediate thickness (I.M.):1-4 mm, and thick: >4 mm).

**Patient Demographics**

All metastatic samples are derived from patients with stage IV disease and have since progressed and died of their disease. All BCC, SCC and thin primary melanomas are derived from patients with no evidence of metastatic disease at the time of surgical excision and there has been no case whereby a patient within this group developed metastatic disease. However, some patients with I.M. and thick primary melanomas had definitive surgical management and subsequently developed either locoregional recurrences or distant metastatic disease. Clinical outcomes and long-term follow-up was not available for most patients in this study due to issues of tissue banking and patient confidentiality policies. Unfortunately, obtaining clinical follow-up information from a different hospital (due to changing positions) was extremely difficult due to HIPPA policies currently in place.
