# Supplementary material for: The gene expression profiles of primary and metastatic melanoma yields a transition point of tumor progression and metastasis
Source: BMC Med Genomics. 2008 Apr 28;1:13. doi: 10.1186/1755-8794-1-13 (PMC2408576; doi:10.1186/1755-8794-1-13)

Gene Ontology and Biological Processes

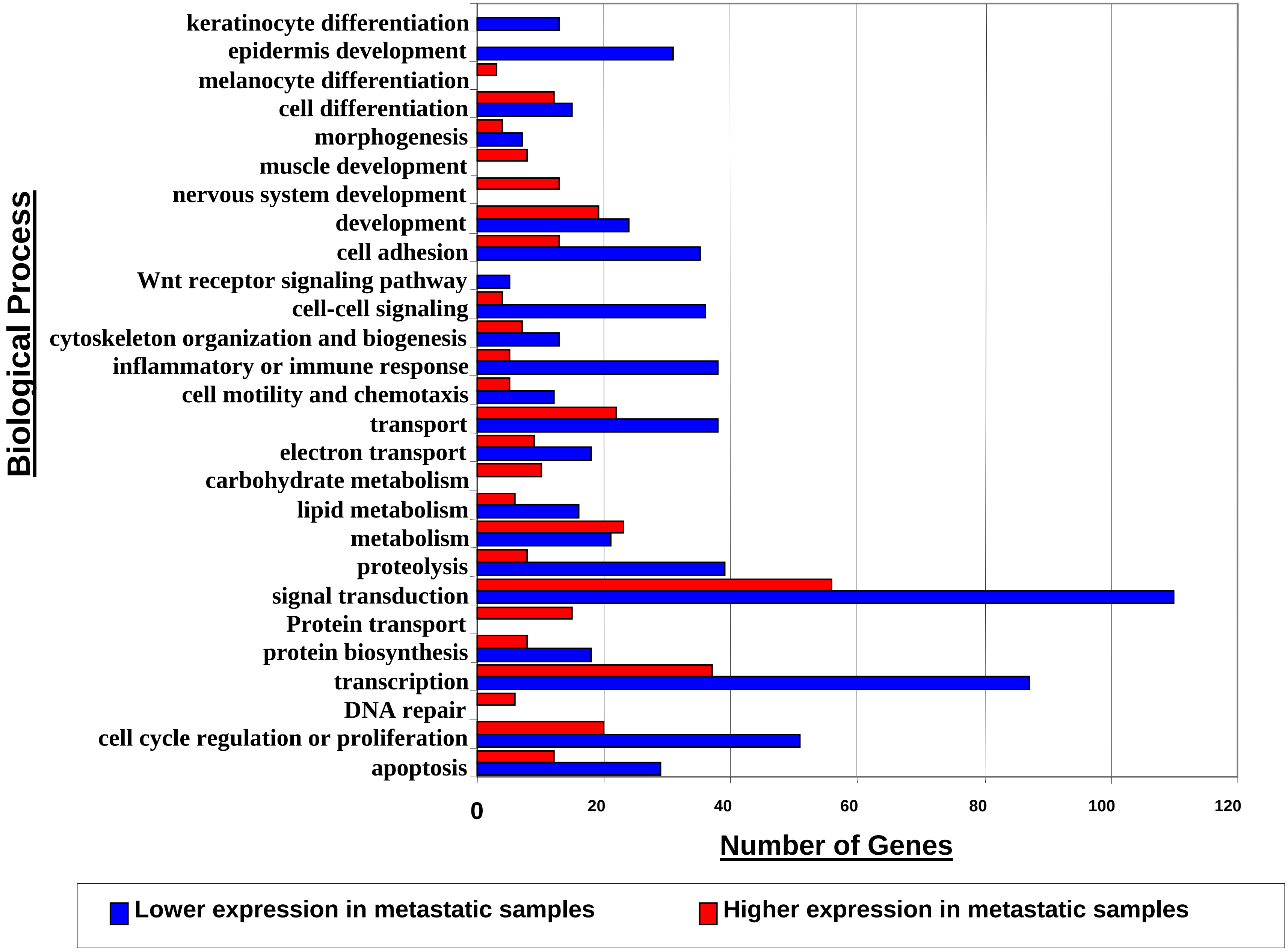

Supplement: Additional file 3 — Gene Ontology and Biological Processes. This file shows the ontological comparisons of various numbers of genes associated with each biological process between primary and metastatic melanoma. [file 1755-8794-1-13-S3.pdf]
